# *Staphylococcus aureus* in Continuous Culture: A Tool for the Rational Design of Antibiotic Treatment Protocols

**DOI:** 10.1371/journal.pone.0038866

**Published:** 2012-07-20

**Authors:** Klas I. Udekwu, Bruce R. Levin

**Affiliations:** Department of Biology, Emory University, Atlanta, Georgia, United States of America; University of Birmingham, United Kingdom

## Abstract

In vitro measures of the pharmacodynamics of antibiotics that account for the factors anticipated for bacteria in infected patients are central to the rational design of antibiotic treatment protocols. We consider whether or not continuous culture devices are a way to obtain these measures. *Staphylococcus aureus* PS80 in high-density continuous cultures were exposed to oxacillin, ciprofloxacin, vancomycin, gentamicin, daptomycin and linezolid. Contrary to results from low density retentostats as well as to predictions of traditional PK/MIC ratios, daily dosing with up to 100× MIC did not clear these cultures. The densities of *S. aureus* in these cultures oscillated with constant amplitude and never fell below 10^5^ CFU per ml. Save for daptomycin “treated” populations, the densities of bacteria in these cultures remained significantly below that of similar antibiotic-free cultures. Although these antibiotics varied in their pharmacodynamic properties there were only modest differences in their mean densities. Mathematical models and experiments suggest that the dominant factor preventing clearance was wall-adhering subpopulations reseeding the planktonic population which can be estimated and corrected for. Continuous cultures provide a way to evaluate the potential efficacy of antibiotic treatment regimes in vitro under conditions that are more clinically realistic and comprehensive than traditional in vitro PK/PD indices.

## Introduction

In recent years there has been a concerted effort to develop antibiotic choice and treatment regimes that are better than those determined empirically. Central to this “rational” design of antibiotic treatment enterprise are PK/PD indices [1], [2]. The numerator of these indices, PK, pharmacokinetics, is a measure of the changes in concentration of the antibiotic during the course of treatment usually estimated in vivo from serum from patients.At least three different measures of the PK are used for these indices; (i) peak antibiotic concentration (C_max_), (ii) time above the minimum inhibitory concentration (MIC), and (iii) area of the antibiotic concentration - time curve (AUC) [3], [4], [5], [6], [7], [8]. The denominator of these indices, PD, pharmacodynamics, is a measure of the relationship between the concentration of the antibiotic and the rate of growth or death of the target bacteria and is almost always estimated in vitro. Although antibiotics are classified as time- or concentration- dependent, the only formal PD parameter used for these indices is the minimum inhibitory concentration, MIC, of that drug for the target pathogen [4], [9], [10].

MICs are estimated in vitro with protocols that are precisely defined for each antibiotic – bacteria combination under culture conditions that are optimal for the action of the drug; low densities of planktonic bacteria (<10^6^) growing exponentially in rich liquid broth under pH and ionic conditions where the drug is most effective [11]. In addition to serving as the denominator of PK/PD indices, MICs are also the dominant parameter employed as a measure of the susceptibility (resistance) of bacteria to antibiotics [11], [12], [13].

It is well recognized that the conditions under which MICs are estimated are unlikely to be met in treated patients and that other elements of the PD of the antibiotic and bacteria could well influence the course of therapy. Included among these other elements are: (i) the functional form of the relationship between the concentration of the antibiotic and the rate of growth/death of bacteria [14]; (ii) the density of the bacterial population [15], [16], [17], [18]; (iii) pH and cation concentrations of the medium or compartment [9], [19], [20], [21]; (iv) the growth rate of the bacteria [22]; (v) non- or slowly- dividing subpopulations of bacteria, persistence [23], [24], [25], [26]; (vi) the physical structure of the bacterial population, e.g. biofilms [27], [28], [29]; vii) intracellular and other refuge-dwelling subpopulations of bacteria [30] and (viii) mortality and delayed replication of antibiotic-exposed bacteria following the elimination of the antibiotic, post-antibiotic effects [20], [31], [32].

How can all of these and other PD factors contributing to the clinical efficacy of antibiotic treatment be addressed at the same time in vitro? A possible way to achieve this more comprehensive measure of the PD of antibiotics and their target bacteria in vitro is by adding these drugs to bacteria maintained in continuous culture devices and following the changes in viable cell densities [9], [33], [34], [35], [36], [37], [38], [39]. With continuous cultures it is possible to evaluate the efficacy of bacteriostatic as well as bactericidal drugs and do so in situations where the concentration of the drug is continually changing. Bacteria in continuous culture can also be used to explore the role of physiologically antibiotic-refractory subpopulations like persisters [23], [24], [25], cells in biofilms, [40], [41], [42], and the contribution of post-antibiotic effects to the dynamics of antibiotic treatment [43]. Continuous culture may also be modified to facilitate study of circulating cell lines containing intracellular bacteria in the future.

In a recent report, we used a mathematical model to provide a theoretical framework to generate hypotheses, design, and interpret the results of in vitro studies of the population dynamics of antibiotic treatment in continuous culture [44]. This model accounts for contribution of the culture density, the physiological state of the bacteria, byproduct resource production by dead cells, antibiotic-refractory persisters, and physically structured subpopulations like biofilms. Here, we use continuous cultures of *Staphylococcus aureus* and antibiotics of six different classes to experimentally evaluate the relative contributions of these different factors to the in vitro population dynamics of antibiotic treatment. These drugs varied considerably in their MICs and rates of kill but with the high concentration, 24 hour dosing regime employed, all six antibiotics were able to reduce the density of viable cells in these cultures and, save for daptomycin, maintain them at levels well below the untreated controls (∼ 10^9^ CFU/ml). On the other hand, even the most effective of the antibiotics employed, gentamicin, was unable to clear these cultures or reduce their densities by more than four orders of magnitude from ∼ 10^9^ CFU/ml of untreated controls. Using the mathematical model presented in [44] and *in vitro* experiments, we explore the processes responsible for these dynamics. Our results suggest that persistence, and in the case of daptomycin a cell-associated decline in the effective concentration of the drug, plays a role in these dynamics. However, the primary factor responsible for preventing clearance of these cultures is the continuous input of bacteria from antibiotic refractory wall-adhering (biofilm) populations. We discuss the implications of these results to the use of continuous culture devices to evaluate the PD of antibiotics for the design of antibiotic treatment protocols.

## Materials and Methods

### Bacterial Strains

All experiments were carried out with the methicillin sensitive *Staphylococcus aureus* clinical isolate PS80, a capsulated (type 8) strain obtained from the American Type Culture Collection (ATCC 27700). Bioassay of antibiotic concentration was assessed with *S.aureus* ATCC 25923.

### Antibiotics

Daptomycin (Cubist Pharmaceuticals, Lexington, MA) and linezolid (Pfizer, New York, NY) was purchased from the Emory University Hospital Pharmacy. Gentamicin, oxacillin, ciprofloxacin and vancomycin were all from Sigma (St. Louis, MO). Stock solutions of these drugs were prepared by dissolving these antibiotics in sterile water or 0.9% (w/v) NaCl, stored at -20°C and used within two weeks of preparation. The concentrations reported are the amount of biologically-active antibiotic in milligrams per litre (mg/L).

### MIC Estimation

Minimum inhibitory concentrations (MICs) of *S.aureus* PS80 and the different antibiotics were estimated by the broth microdilution method recommended by Clinical and Laboratory Standards Institute (CLSI) guidelines [11], but incubating the cultures at 37°C (rather than 35°C) and slight modification; 25% rather than 50% dilutions of the antibiotics were used for enhanced resolution and optical density (OD_630_) to monitor growth. The values reported represent the lowest antibiotic concentration displaying no growth (The OD_630_ (18h) ≤ OD_630_ (0h).

### Batch Culture Conditions and Media Preparation

Bacterial cultures were incubated overnight at 37 °C with shaking at 200 rpm in cation-adjusted Mueller-Hinton II (MHII) medium alone (supplemented with 50 mg/L CaCl_2_ for experiments with daptomycin). Time kill assays were performed under aerobic conditions with constant agitation at 37 °C. For batch culture exposure, we used 20× MIC (ciprofloxacin & linezolid), 50× MIC (daptomycin & gentamicin) or 100× MIC (oxacillin & vancomycin) of antibiotic. To test for media nutritional quality, the medium was filtrated of cells by passing through a 0.22 micron filter and 1×10^5^, 1×10^6^, and 1×10^7^ cells were added. The maximum exponential growth rates were estimated from OD_630_ measurements obtained during growth at 37°C with aeration. The maximum cell densities were estimated from colony forming units plated after overnight growth in the respective media.

### Continuous Cultures

The continuous culture devices used in this study were of homemade-design and are thoroughly described in www.eclf.net. The volume of culture in the vessels ranged from 20 to 25 ml and the cell generation time was adjusted by changing the speed of the peristaltic pumps. The rate of flow of medium into (and waste from) these vessels ranged from 0.6 ml per hour to 8.6 ml per hour for dilution rates, ∼ 0.04, 0.18 and 0.43 hr^−1^. In reporting these results and calculating the rate of antibiotic decay, we used the approximate dilution rates, 0.04, 0.20, and 0.40. Experiments were not initiated until the densities of bacteria in successive samples were equal and these cultures were at the density equilibrium (more than four volume changes). The culture vessels were maintained under negative pressure and aerated as well as vigorously mixed by air sucked into vessels through 0.45 µm filters. Bacteria and antibiotics were introduced and sampled from the screw-top side arm in the culture vessel with minimal perturbation of the vacuum, see www.eclf.net/chemostat.

### Bioassay Estimation of Residual Antibiotic Concentration

Medium from the desired time point was taken from the culture vessel, centrifuged and passed through 0.45 µm filters prior to bioassay estimation of drug concentration using the broth microdilution described in [15], the standard *S.aureus* ATCC 25923, and comparing to a standard containing fresh antibiotic.

### Assessment of Physiological State during Antibiotic Exposure

During the six-day treatment, three hours after each pulse of 100× MIC (Time points 3, 51, and 123 hours), we transferred an aliquot of the surviving population to 12 well FALCON™ plates and incubated for an additional 3 hours. Controlling for bioassay-estimated loss of antibiotic in the medium, these were compared to an equivalent density of naïve exponential cells exposed for three hours in the filtrated, bioassayed supernatant from each culture vessel at the particular time point. Comparison was then made between the surviving densities of naïve and pre-exposed (sophisticated) cells in these batch cultures as well as the densities in the corresponding continuous cultures at 6, 54, and 126 hours respectively.

### Estimation of the Density of Bacteria Released from the Walls

Immediately after the termination of the 6-day treatment period experiments, the medium in the 0.2 hr^−1^ flow rate continuous cultures with ciprofloxacin, daptomycin and gentamicin was removed. The culture vessels were washed by adding equal volumes of 1% PBS, sonicated for one minute and the liquid removed. This procedure was repeated twice after which an equal volume of MHII medium was added to the vessel and the culture sampled. After 5 minutes of sonication at 56 KHz, the viable cell density was estimated. The density of cells in the residual wall population was calculated as the difference between the pre-sonicated, and the sonicated samples.

## Results

### Minimum Inhibitory Concentrations

Using the modified CLSI serial dilution protocol [11], the MICs estimated for the antibiotics used in these experiments were, respectively 0.5 mg/L for Ciprofloxicin, Daptomycin, and Gentamicin, 1.0 mg/L for Linezolid and Vancomycin, and 0.4 mg/L for Oxacillin.

### Antibiotic exposure of *S. aureus*in continuous culture

#### Single dose experiments

**Figure 1:**
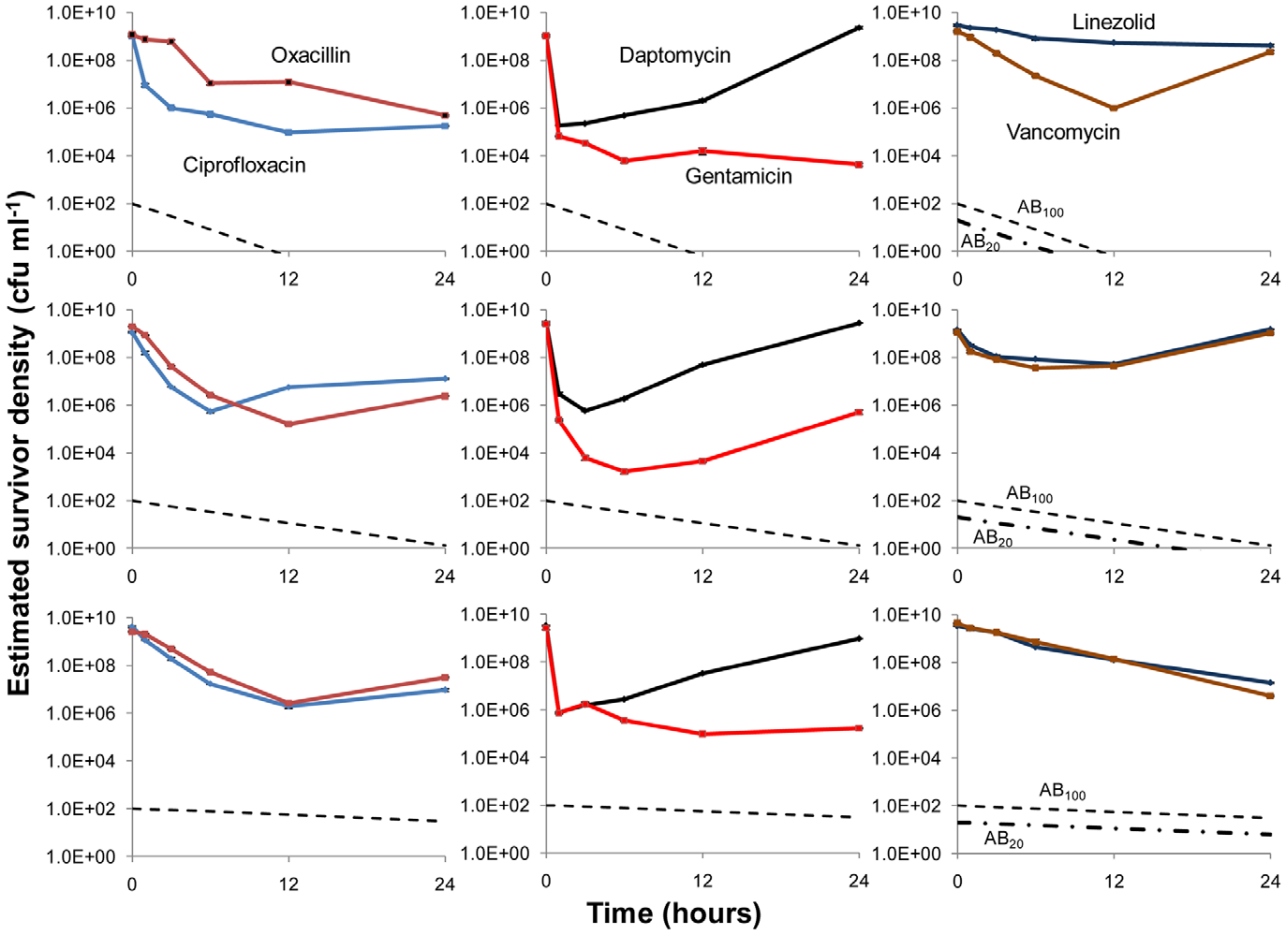
Changes in viable cell density and theoretically predicted antibiotic concentrations in antibiotic-treated continuous cultures maintained at three different dilution rates: Fast w ∼ 0.4 (top row); moderate w ∼ 0.2 (middle row); and slow, w ∼ 0.04 (bottom row) upon treatment with single antibiotic doses added at time 0. Save for linezolid, which because of its lower solubility was added at 20× the MIC, the initial concentration of the antibiotics were approximately 100× MIC. The changes in the concentrations of the antibiotics in these figures, the broken lines, are calculated from equation (1) under the assumption that these concentrations decline exponentially at rates equal to that of flow.

In Figure 1, we present the results of experiments in which single doses of antibiotics were added to continuous cultures at equilibrium maintained at different dilution rates and the viable cell densities estimated from CFU data. The change in the concentration of the antibiotic in this figure is that calculated under the assumption that the concentration of the antibiotic is declining exponentially at a rate equal to the flow rate, 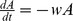 or the concentration at a given time t,
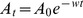
(1)where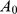 is the initial concentration (see Appendix S1).

At the highest dilution rate, w = 0.4 hr^−1^ the anticipated concentration of all the antibiotics would be less than the MIC by 12 hours for all antibiotics save for linezolid, where the concentration would fall below the MIC by 8 hours. At the intermediate dilution rate, w = 0.2 hr^−1^, save for linezolid the anticipated concentrations of the antibiotics remain above the MIC for nearly 24 hours, ∼16 hours for linezolid. As measured by the maximum extent of antibiotic-mediated killing, at all dilution rates gentamicin is the most effective of these six antibiotics and ciprofloxacin second, although at the lower dilution rates oxacillin appears to kill to the same extent as ciprofloxacin. At the highest but not lower dilution rates, vancomycin also appears to be as effective as ciprofloxacin. While initially daptomycin rapidly reduces the viable cell density, this killing effect is transient. By 24 hours, the population returns to its untreated level at all dilution rates, including those in lower dilution rate continuous cultures where high concentrations of this drug are anticipated to be present throughout the experiment. Although the concentrations of the antibiotics are anticipated to fall below the MIC by or before 12 hours in the fast continuous cultures, save for vancomycin and daptomycin the viable cell densities did not return to their pre-treatment level by 24 hours.

#### Multiple treatment experiments

**Figure 2:**
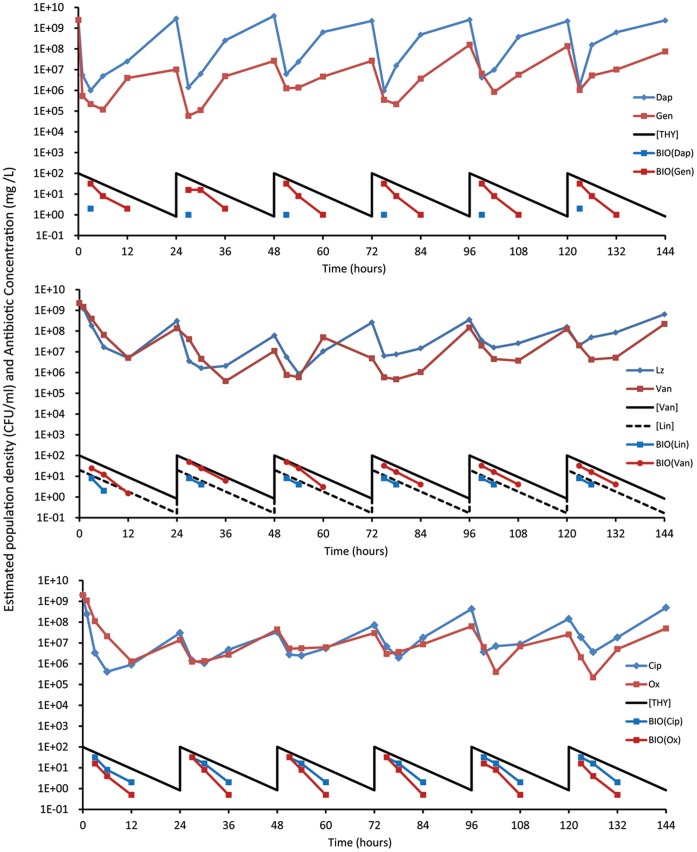
Changes in the viable cell densities (CFU/ml) of *S. aureus* and concentrations of antibiotics (X MIC) in continuous cultures treated every 24 hours with antibiotics of six classes. The dilution rate of these continuous cultures was w = 0.2 h^−1^. Save for linezolid, which was administered at 20× MIC, the daily treatment concentrations of these drugs were 100× their respective MICs. The changes in the concentrations of the antibiotics in the solid line are those calculated from equation (1). The antibiotic concentrations estimated from the bioassay are noted in the lines with points. Top panel daptomycin (1.0±0.32×10^7^), gentamicin (1.9±0.67×10^6^); Middle panel linezolid (2.2±0.83×10^7^) and vancomycin (8.4±4.1×10^6^); Bottom panel, ciprofloxacin (6.3±1.6×10^6^) and oxacillin (4.5±1.3×10^6^). The numbers in parentheses are the daily estimates of the harmonic mean densities of viable cell (CFU data) of the continuous cultures treated with the noted antibiotic and the standard errors of those estimates over the 6 days of “treatment”. In Table S1 we present bioassay estimates of the concentrations of these antibiotics.

In Figure 2, we present the results of experiments where initially equilibrium continuous cultures running at dilution rate *w* = 0.2 hr^−1^ are “treated” every 24 hours for six days. Particularly striking is the observation that despite daily dosing with 100× MIC (20× for linezolid), the viable cell densities do not continue to decline or fall below 10^5^ cells per ml. (approximately four orders of magnitude less than the antibiotic-free equilibrium density). Save for daptomycin, the maximum viable cell densities in these treated continuous cultures remained one to two orders of magnitude lower than the untreated control cultures. Although daptomycin is highly effective in killing *S. aureus* shortly after its introduction, within 24 hours the populations return to their pre-treatment levels and already start to recover when the anticipated concentration of the drug is more than 50× MIC. As measured by the harmonic mean of the number of viable cells over the period of treatment, gentamicin is the most effective antibiotic. Oxacillin, ciprofloxacin and vancomycin are next in this hierarchy, followed by daptomycin and linezolid. Of some interest in this regard is that linezolid is dosed at 0.2 to 0.4 times the relative MIC of the other antibiotics and this drug is supposed to be bacteriostatic, rather than bactericidal. Although not run simultaneous as in this figure or under precisely the same conditions, this continuous dosing experiment was performed at least once more for each of these antibiotics. While there were some differences in the average densities of these cultures, the basic results were repeated. In none of these experiments were the bacteria cleared, their densities oscillated and did not decline to levels much less than 10^4^ (Data not shown).

### Hypotheses and their Tests

Why do the densities of viable bacteria in these periodically treated continuous culture populations oscillate around a constant level rather than continue to decline as a result of daily “treatment” with antibiotics? In our theoretical study of these dynamics [44] we considered three mechanisms that could delay or prevent clearance, having precluded contamination as a cause. The first is persistence, a subpopulation of cells, *P*, that are physiologically refractory to antibiotics because they are either not dividing or dividing very slowly [24], [45], [46]. The second is an antibiotic refractory wall (biofilm) population, *B*, which is washed out at a lower rate than the planktonic *N* and *P* populations. The third is that the effective concentrations of the antibiotics in these cultures are substantially less than that anticipated from washout-dependent flow alone.

**Figure 3:**
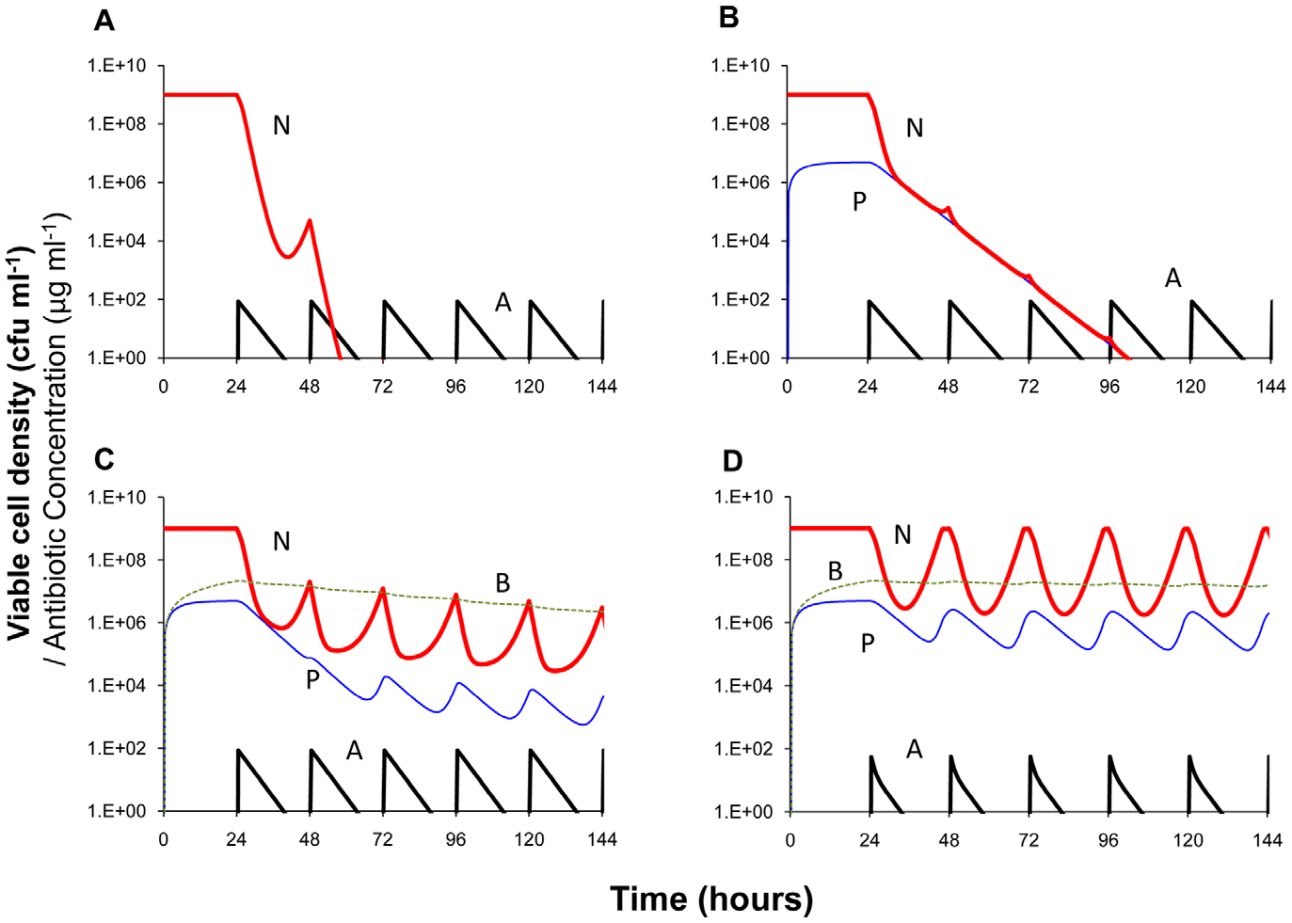
Simulation of antibiotic treatment of bacteria in continuous culture (numerical solutions to the model used in [44]; changes in viable cell density and antibiotic concentration. 100× MIC of antibiotic administered every 24 hours starting on the second day, 24 hours. Standard parameters, C = 500, V_max_ = 1, V_min_ = −1, κ = 1, km = 0.25, e = 5×10^−7^, M_min_ = 1, M_max_ = 40, ka = 10^−7^, f = 0, pd = 0, cr = 0.9, dx = 0.1, dv = 0, dk = 0,. A - No persistence or wall population. B - An antibiotic refractory persister, P, population, V_maxP_ = 0.001, V_minP_ = −0.001, f_NP_ = f_PN_ = 0.001. C - An antibiotic refractory persister, P, and wall population, B, V_maxB_ = 0.001, V_minB_ = −0.1, f_NB_ = 0.001, f_BN_ = 0.01, w_B_ = 0.001. D - An antibiotic persister and wall population with density-dependent decay in the effective concentration of the antibiotic, dv = 10^−9^. The definitions and dimensions of these parameters are presented in the text and Table 1 of [44] where the model is described in detail. Copies of the Berkeley Madonna™ program used for these simulations can be downloaded from www.eclf.net/programs.

In Figure 3, we use a numerical solution to the differential equation model (computer simulations) in [44] to illustrate the contribution of persisters, wall (biofilm) populations, and density-dependent decay of the antibiotic to the changes in viable cell density anticipated for continuous culture experiments similar to those in Figure 2. In these simulations we assume a bactericidal antibiotic and use population growth parameters in a realistic range for *S. aureus* maintained in MHII. The parameters for the persister and wall populations, their generation, loss and antibiotic susceptibility are assumed and chosen to illustrate their effects.

With these parameters, in the absence of a persister or wall population, we would anticipate clearance of the culture by the second day (Figure 3A). Persistence alone would reduce the rate at which the population is cleared, but would not account for the seemingly constant amplitude continuous oscillations in viable cell density observed (Figure 3B). If however, there is an antibiotic-refractory wall population (Figure 3C), the total population density continues to oscillate over the full period of treatment and only slowly approaches clearance. Assuming density-dependent reductions in the concentration of the antibiotic, these oscillations are maintained over the treatment period with seemingly constant period and amplitude. Moreover, with this density-dependent decay in the effective concentration of the antibiotic, between doses the viable cell density of the planktonic population returns to its pretreatment level (Figure 3D).

Based on the results of these simulations, we postulate that the dominant factor responsible for the failure to clear the bacteria in these continuously flowing cultures is an antibiotic-refractory, wall-adhering population that continually reseeds the planktonic bacteria being sampled. We also postulate that the daily resurrections of the daptomycin-treated population can be attributed almost wholly to a density-dependent decay in its effective concentration.

**Figure 4:**
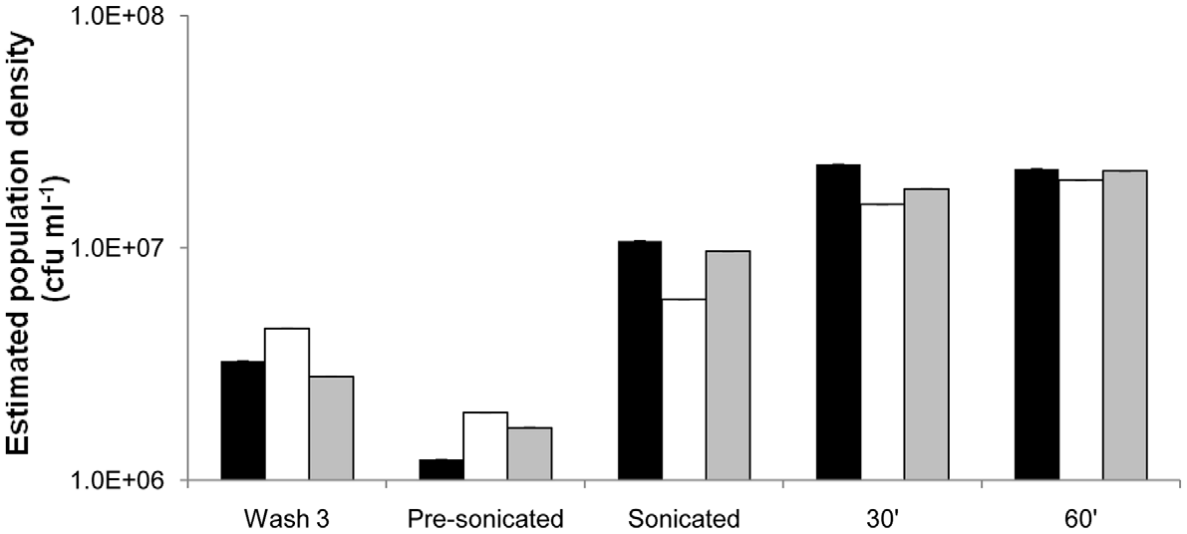
Mean viable cell densities of bacteria released from the walls of the culture vessels following 6 days of daily dosing with ciprofloxacin (black), daptomycin (white) and gentamicin (shaded). Wash 3 - densities of bacteria in PBS following the third wash. Pre-sonicated - densities of bacteria in MHII added to the vessels immediately prior to sonication but after washing. Sonicated - densities of these cultures in MHII after 5 minutes of sonication. 30′ and 60′ – respectively, densities estimated in MHII 30 and 60 minutes after MHII addition and sonication. Three independent estimates of densities were made for each sample. All standard errors were less than 0.1% of their respective means.

To test for the presence of an antibiotic refractory wall population of adequate densities to explain the Figure 2 results, we removed the planktonic cells from the culture vessels, washed them thrice in equal volumes of PBS. We then added MHII medium and estimated the density of viable cells before and after sonicating the vessel to release bacteria adhering to the walls. The results of these experiments for the vessels treated with ciprofloxacin, daptomycin and gentamicin are presented in Figure 4.

We interpret these results as support for the hypothesis that a refractory wall population is responsible for the failure of these antibiotics to clear these continuously flowing cultures. Considering the density of planktonic bacteria estimated immediately following sonication and after 30 and 60 minutes of incubation in MHII, for all three of these antibiotics, the density of suspended viable bacteria removed from the wall is between 5×10^6^ and 2×10^7^ CFU/ml.

#### Antibiotic decay

To determine whether the concentrations of the antibiotics in these cultures are in the range anticipated by exponential decline at a rate equal to the flow, we estimated the ‘MIC’s of these filtrates taken at 3, 6, and 12 hours after drug addition, using low densities (∼10^5^ cells per ml) of *S. aureus* PS80. The results of these experiments are presented in Figure 2 and also in Table S1.

Save for daptomycin, the bioassay estimates of antibiotic concentration are usually between a factor of 2 and 4 times less than the predicted concentrations of these drugs. Remarkably, by 3 hours the biologically active concentration of daptomycin is between 25 and 50 times less than that predicted from the exponential decay model. By six hours the cultures appear to be effectively free of this drug. We interpret this result as support for the hypothesis that resurrection of the daptomycin treated cultures to pre-treatment levels each day can be attributed to the density-dependent uptake of this drug in these cultures. In the absence of bacteria there was no evidence for a decline in the effective concentration of this drug in MHII [15].

This model and these data suggest that the primary reason for the failure of antibiotics to clear the continuous culture is an antibiotic-refractory wall population. Since the colonies of bacteria isolated at the end of these experiments had similar MICs to the treating drugs as the original cells, we can exclude inherited resistance in the majority population.

#### Persistence

The results of our other experiments indicate that an antibiotic refractory wall (biofilm) population is not the sole physiologically resistant subpopulation contributing to these dynamics. The planktonic bacteria in the antibiotic-treated continuous culture are collectively less susceptible to the majority of these antibiotics than naïve cells, presumably because of antibiotic enrichment of persistent subpopulations. The evidence for the bacterial populations in these exposed continuous cultures being more physiologically refractory to antibiotics than unexposed cells, comes from comparing the results of time kill experiments carried out on pre-exposed, and non-previously-exposed populations of *S.aureus*.

For the pre-exposed (sophisticated) populations, planktonic cells were removed three hours after antibiotic addition to the continuous cultures and incubated under batch conditions for an additional 3 hours, at which time the viable cell density of these bacteria was estimated. For the unexposed (naïve) bacteria exponentially growing cells from antibiotic-free batch cultures were introduced into sterile filtrates from the 3-hour, antibiotic-treated continuous culture and their survival estimated from viable cell densities after 3 hours of exposure. The densities of “naïve” cells added were adjusted to be approximately the same as those in the corresponding experiment with previously exposed (sophisticated) bacteria. It should be noted that since the densities of bacteria in the continuous cultures 3 hours after treatment were more than two orders of magnitude less than that of antibiotic free cultures at the density equilibrium, the MHII medium was essentially at full concentration.

**Figure 5:**
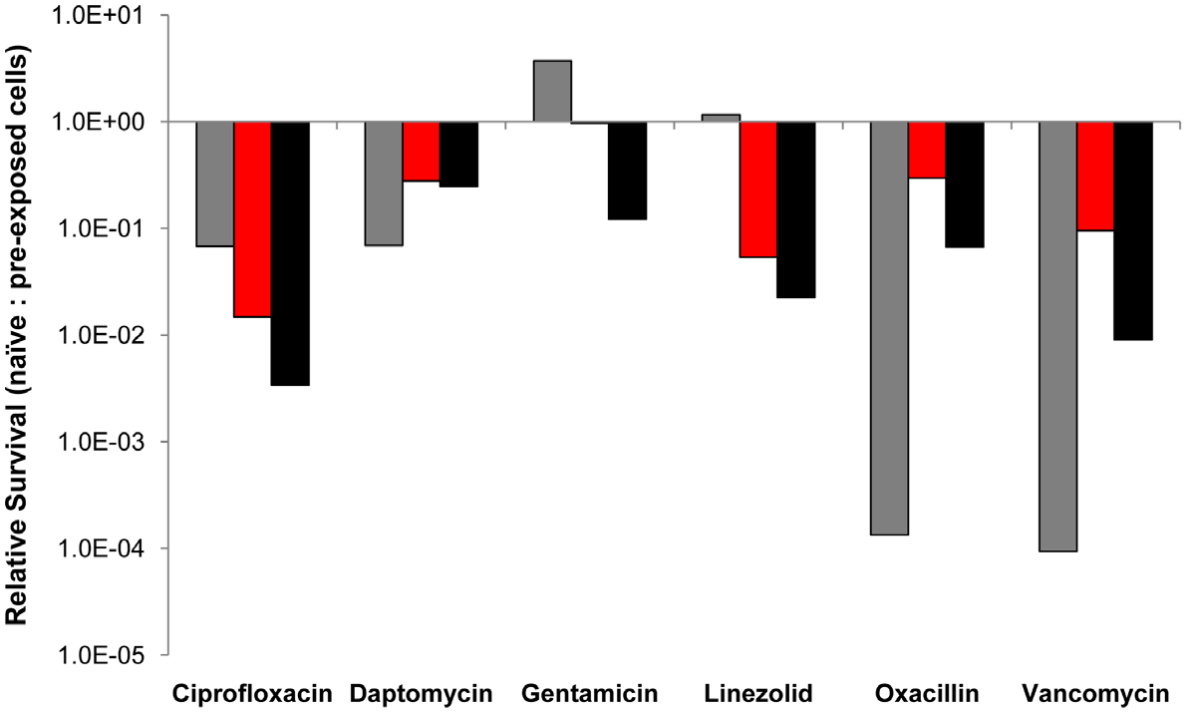
Relative survival of unexposed (naïve) to previously antibiotic-exposed (sophisticated) *S. aureus* PS80 after three hours of exposure in antibiotic-containing filtrates from the treated continuous culture (Figure 2). The sophisticated bacteria and filtrates used for these experiments were taken at 3 hours (gray), 51 hours (red), and 123 hours (black). The biologically active concentrations of antibiotics are reported in Table S1. Data summarized from Figure S1.

The results of these experiments are presented in a composite figure, where we plot the ratio of the densities of surviving naïve to sophisticated cells after three hours of exposure to these drugs (Figure 5). If the sophisticated and naïve bacteria were equally susceptible this ratio would be 1.0. If the pre-exposed population were less susceptible to the antibiotic than naïve cells, the ratio would be less than 1.0.

By this criterion, the bacteria exposed to ciprofloxacin, daptomycin, oxacillin, vancomycin and linezolid are clearly more refractory to these antibiotics than naïve cells. This does not appear to be case for gentamicin. It should be noted that although linezolid is supposed to be bacteriostatic, at the concentration present at 3 hours (about 8× MIC – see Table S1) this drug is bactericidal and seemingly more so for the naïve bacteria than those that previously encountered this drug. For interested readers, the results of the naïve and pre-exposed experiments from whence this composite figure was derived are separately plotted in Figure S1.

## Discussion

As complex as continuous culture populations of bacteria may be, they barely come close to mimicking the temporal, spatial, nutritional, and physiological heterogeneity of bacteria in infected humans or other mammals. On the other hand, these continuous cultures provide more comprehensive (than batch cultures) and, for infected patients more realistic, in vitro measures of the pharmacodynamics of antibiotics and bacteria than those currently used for rational design recommendations of antibiotic treatment [1], [2]. The bacteria in these cultures are at the high densities, and include physiologically refractory planktonic and biofilm subpopulations, that could be anticipated for many real bacterial infections. Moreover, as would be expected in treated patients, the concentrations of the antibiotics are in a continuous state of flux.

**Figure 6:**
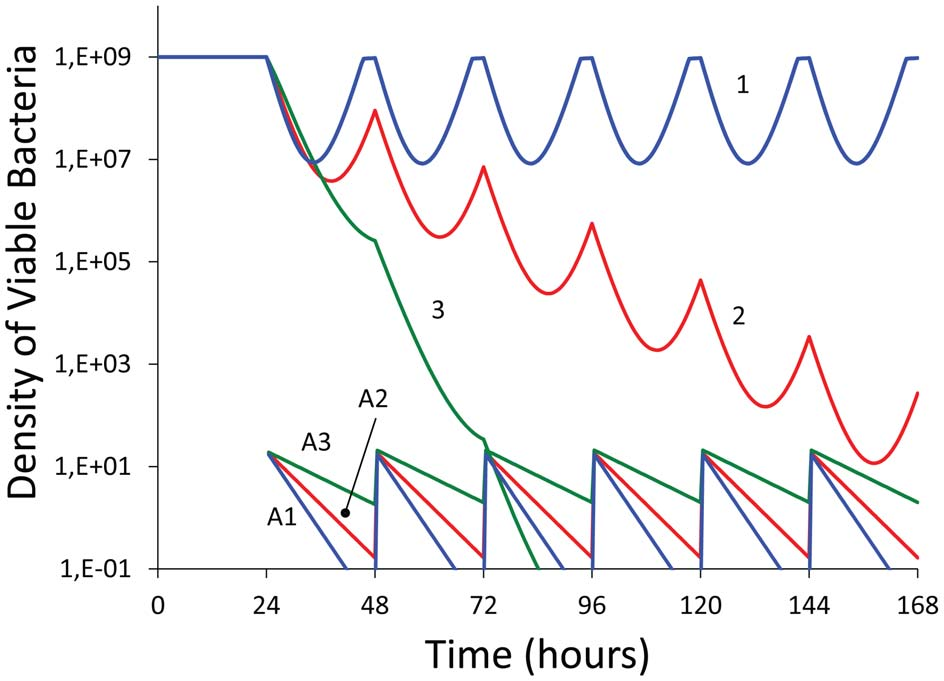
Simulation of antibiotic treatment of bacteria in continuous culture under optimal conditions, no biofilm or persister subpopulations with constant Hill function parameters; numerical solutions to the equations of the model used in [44]. Changes in antibiotic concentration (A1, A2 & A3) and viable cell density of planktonic bacteria (1, 2, and 3). In all simulations the maximum and minimum hourly growth/death rates are respectively, V_MAX_ = 1, V_MIN_ = −0.5, the Hill coefficient κ = 1.0 and the MIC = 2 mg L^−1^. Every 24 hours, 20 mg/L of the antibiotic was added and is the Peak concentration for all runs. The flow rates and PK/MIC ratios for the Time > MIC and AUC are respectively: For w = 0.3, **3.85** and **33.3**; for w = 0.2, **5.75** and **49.6**; and for w = 0.1, **11.5** and **90.9**, The equations for calculating the PK element of these indices is noted in Appendix S1 under the assumption that the only source of decline in the concentration of the antibiotic is the flow rate, δ = w.

The results of these experiments with *S. aureus* in continuous cultures could not be predicted from PK/PD indices based on MICs estimated under the conditions specified by CLSI protocol [11] or traditional time kill data, obtained under optimal conditions. To illustrate this we use our model and consider a hypothetical bactericidal antibiotic that is less effective than those used in our experiments, i.e. has a higher MIC and lower rate of kill (the V_MIN_ parameter in the Hill function). In these simulations we also use a lower concentration at each dose, 10× MIC, rather than the 100× or 20× MIC than employed in our experiments, thereby more reflective of doses employed clinically. The results of these simulations are presented in Figure 6. The PK/MIC ratios (indices) for these “treatments” are noted in the legend to this figure. The order of these indices is that anticipated for a commonly employed PK/PD recommendation for treatment, the higher the ratio the greater the rate of clearance.

Contrary to the prediction of this model, clearance was never observed in the analogous continuous culture experiments depicted in Figure 2 where antibiotic concentrations and PD measures of antibiotic efficacy were more favorable for clearance than those considered in these simulations. While the densities of viable cells in these continuous cultures declined immediately after dosing, they rose before the subsequent dose was administered and never fell below 10^5^. Although these drugs varied in their efficacies as measured by the CLSI MICs and maximum rates of kill (data not shown), the harmonic (or arithmetic) mean densities of these cultures were not very different. By this average density criterion, linezolid, which is considered to be bacteriostatic, was almost as effective as gentamicin, which is highly bactericidal. There was however, some disparity in linezolid treated cultures with higher than normal variance (1.5 logs) in samples from the first 12 hours of exposure. This may be due to the low solubility of the drug and the cultures thus demanding large volumes (up to 2 ml) of antibiotic to attain 20× MIC concentrations. Despite this, the cycling behavior of the treated population was reproducible in replicate experiments. Save for daptomycin, the sustained densities of bacteria in these antibiotic treated cultures remained at least an order of magnitude lower than that anticipated for antibiotic free controls. Stated another way, for five of the antibiotics these continuous cultures were limited by the antibiotics rather than resources or nutrients, which remained at same concentrations required for optimal growth.

One virtue of using “treated” continuous cultures for vitro explorations of the potential clinical efficacy of antibiotics is that they provide a way to identify and evaluate the contributions of factors affecting the PDs of antibiotics that would not be detected with traditional in vitro estimation procedures. The results of our experiments suggest that the most important of these factors are phenotypically refractory subpopulations and particularly those in wall-adhering biofilms. The importance of biofilm subpopulations in preventing the eradication of genetically susceptible *S. aureus* in antibiotic-treated continuous cultures was observed some time ago [47]. The results of our experiments add generality to this observation, demonstrating that a biofilm confounding effect would be anticipated even when, unlike in these earlier studies, multiple doses of antibiotics of six different classes are used and the concentrations of the drugs remain significantly above the MIC for most if not all of the time between doses.

Not anticipated from traditional low-density estimates of MICs is the rapid reduction in the effective concentration of daptomycin observed in our experiments. As a consequence of this decline, the bacterial population returns to a density anticipated for antibiotic free cultures each day. This may be attributed to irreversible and non-productive uptake of the cyclic peptide [15]. These in vitro results certainly raise the question about whether this density-dependent loss in effective concentration contributes to some of the treatment failures observed when this cyclic peptide is used for treating *S. aureus* infections e.g. [48], [49].

In this report, we have restricted our experiments elucidating the factors contributing to the pharmaco-, and population dynamics of antibiotic “treatment” of continuous culture populations of *S. aureus.* As such, we do not take into account intracellularly localized, or other non-planktonic populations and intracellular subpopulations of *S.aureus* have been implicated in several recurrent infection types and a macrophage cell line (reviewed in [30]). These would warrant inclusion in future versions of our model (work in progress) that can account for such cells and their impact on the PD/PK. We have not estimated the exact contribution of biofilm seeding into the media but from the densities estimated in the washes, these adherent populations appear to be on the order of 10^5^ cfu per ml. Accordingly, the rate at which this population would produce planktonic cells may be estimated by repeated sampling of a bubbling culture post nutrient removal and device washing. Although beyond the scope of this study, it would seem that this experimental system could also be used to further develop and evaluate different methods to increase the efficacy of treatment. Additionally, while empirically determined treatment regimen are the ultimate goal, results obtained from such in vitro PK/PD studies will serve well to provide testable hypotheses for both optimal choice and length of antibiotic treatment. The present results suggest that one of the most important target for these interventions are the wall-adhering biofilms that prevent clearance. A number of agents have been identified that can either prevent the formation of or disrupt established biofilms. Included among them are D-amino acids, [50], zaragozoic acid [51], metabolites that enter upper glycolosis, like glucose, mannitol, pyruvate [52], and even bacteriophage [53]. It would be of some interest to ascertain the extent to which these and other interventions improve the course of therapy, i.e. reduce the level of the oscillations and ideally, clear these cultures. Inclusion of an intracellular subpopulation would further serve to refine this model contributing to the phenotypically refractive population epitomized by the biofilm compartment alone in this study.

## Supporting Information

Figure S1 **Density of surviving cells after three hours when equal densities of bacteria from the continuous culture with antibiotics; pre-exposed (P-E) (“Sophisticated”) cells and unexposed (“Naïve”) cells exposed to filtrates of medium taken from 0.2 hour flow rate continuous culture with the drug.** The gray, red and black columns are respectively the viable cell densities of sophisticated and naïve *S aureus* in filtrates of treated continuous culture removed at 3, 51 and 123 hours, respectively (see the text).
(TIF)

Table S1 **Concentrations of antibiotics at different times during the course of antibiotic treatment of the continous cultures in** **Figure 2** **.** Bioassay concentrations given as multiples ofthe CLSI estimates of the MICs of these antibiotics.
(DOCX)

Appendix S1 **Mathematical equations describing the pharmacokinetics, PK, of antibiotics in continuous cultures.**
(DOCX)
