# Supplementary figures and images for: Staphylococcus aureus in Continuous Culture: A Tool for the Rational Design of Antibiotic Treatment Protocols
Source: PLoS One. 2012 Jul 20;7(7):e38866. doi: 10.1371/journal.pone.0038866 (PMC3401188; doi:10.1371/journal.pone.0038866)

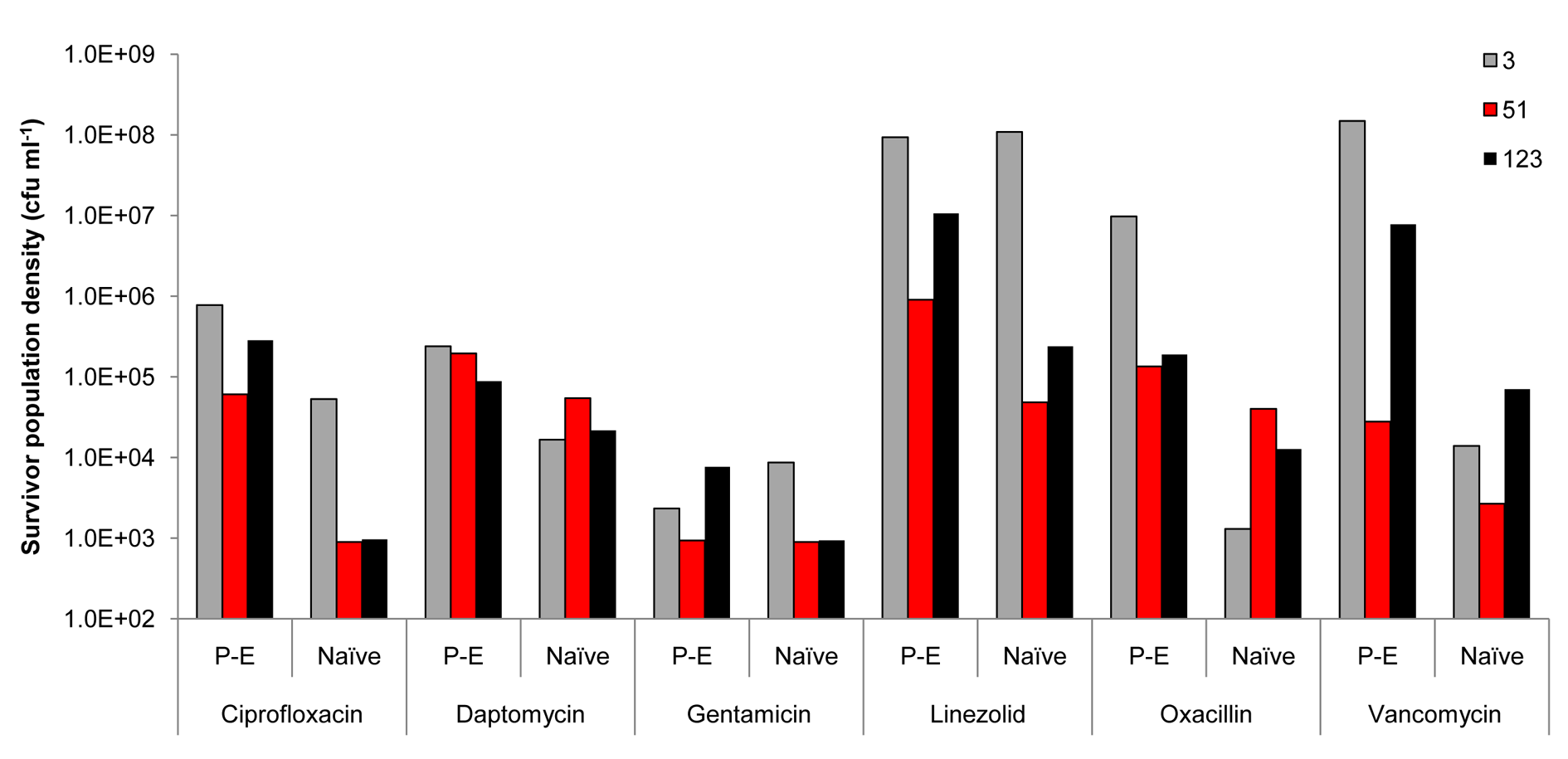

Supplement: Figure S1 — Density of surviving cells after three hours when equal densities of bacteria from the continuous culture with antibiotics; pre-exposed (P-E) (“Sophisticated”) cells and unexposed (“Naïve”) cells exposed to filtrates of medium taken from 0.2 hour flow rate continuous culture with the drug. The gray, red and black columns are respectively the viable cell densities of sophisticated and naïve S aureus in filtrates of treated continuous culture removed at 3, 51 and 123 hours, respectively (see the text). (TIF) [file pone.0038866.s001.tif]
