# Supplementary material for: Staphylococcus aureus in Continuous Culture: A Tool for the Rational Design of Antibiotic Treatment Protocols
Source: PLoS One. 2012 Jul 20;7(7):e38866. doi: 10.1371/journal.pone.0038866 (PMC3401188; doi:10.1371/journal.pone.0038866)
Supplement: Table S1 — Concentrations of antibiotics at different times during the course of antibiotic treatment of the continous cultures in Figure 2 . Bioassay concentrations given as multiples ofthe CLSI estimates of the MICs of these antibiotics. (DOCX) [file pone.0038866.s002.docx]

**Table A1.** Residual concentration of antibiotics during the 6 day in vitro treatment assay.

|  | **Antibiotic Concentration***  **(# x MIC)** | | | | | | | | | | | |
| --- | --- | --- | --- | --- | --- | --- | --- | --- | --- | --- | --- | --- |
|  | **[Ciprofloxacin]** | | **[Daptomycin]** | | **[Gentamicin]** | | **[Linezolid]** | | **[Oxacillin]** | | **[Vancomycin]** | |
| Time (h) | THY | BIO | THY | BIO | THY | BIO | THY | BIO | THY | BIO | THY | BIO |
| 0 | 100.0 |  | 100.0 |  | 100.0 |  | 20.0 |  | 100.0 |  | 100.0 |  |
| 3 | 54.9 | 32 | 54.9 | 2 | 54.9 | 32 | 11.0 | 8 | 54.9 | 16 | 54.9 | 24 |
| 6 | 30.1 | 8 | 30.1 | 0 | 30.1 | 8 | 6.0 | 2 | 30.1 | 4 | 30.1 | 12 |
| 12 | 9.1 | 2 | 9.1 | 0 | 9.1 | 2 | 1.8 | 0 | 9.1 | 0.5 | 9.1 | 1.5 |
| 24 | 0.8 |  | 0.8 |  | 0.8 |  | 0.2 |  | 0.8 |  | 0.8 |  |
| 24 | 100.8 |  | 100.8 |  | 100.8 |  | 20.2 |  | 100.8 |  | 100.8 |  |
| 27 | 55.3 | 32 | 55.3 | 1 | 55.3 | 16 | 11.1 | 8 | 55.3 | 32 | 55.3 | 48 |
| 30 | 30.4 | 16 | 30.4 | 0 | 30.4 | 16 | 6.1 | 4 | 30.4 | 8 | 30.4 | 24 |
| 36 | 9.1 | 2 | 9.1 | 0 | 9.1 | 2 | 1.8 | 0 | 9.1 | 0.5 | 9.1 | 6 |
| 48 | 0.8 |  | 0.8 |  | 0.8 |  | 0.2 |  | 0.8 |  | 0.8 |  |
| 48 | 100.8 |  | 100.8 |  | 100.8 |  | 20.2 |  | 100.8 |  | 100.8 |  |
| 51 | 55.3 | 32 | 55.3 | 1 | 55.3 | 32 | 11.1 | 8 | 55.3 | 32 | 55.3 | 48 |
| 54 | 30.4 | 16 | 30.4 | 0 | 30.4 | 8 | 6.1 | 4 | 30.4 | 8 | 30.4 | 24 |
| 60 | 9.1 | 2 | 9.1 | 0 | 9.1 | 1 | 1.8 | 0 | 9.1 | 0.5 | 9.1 | 3 |
| 72 | 0.8 |  | 0.8 |  | 0.8 |  | 0.2 |  | 0.8 |  | 0.8 |  |
| 72 | 100.8 |  | 100.8 |  | 100.8 |  | 20.2 |  | 100.8 |  | 100.8 |  |
| 75 | 55.3 | 32 | 55.3 | 1 | 55.3 | 32 | 11.1 | 8 | 55.3 | 32 | 55.3 | 32 |
| 78 | 30.4 | 16 | 30.4 | 0 | 30.4 | 8 | 6.1 | 4 | 30.4 | 8 | 30.4 | 16 |
| 84 | 9.1 | 2 | 9.1 | 0 | 9.1 | 1 | 1.8 | 0 | 9.1 | 0.5 | 9.1 | 4 |
| 96 | 0.8 |  | 0.8 |  | 0.8 |  | 0.2 |  | 0.8 |  | 0.8 |  |
| 96 | 100.8 |  | 100.8 |  | 100.8 |  | 20.2 |  | 100.8 |  | 100.8 |  |
| 99 | 55.3 | 32 | 55.3 | 1 | 55.3 | 32 | 11.1 | 8 | 55.3 | 16 | 55.3 | 32 |
| 102 | 30.4 | 16 | 30.4 | 0 | 30.4 | 8 | 6.1 | 4 | 30.4 | 8 | 30.4 | 16 |
| 108 | 9.1 | 2 | 9.1 | 0 | 9.1 | 1 | 1.8 | 0 | 9.1 | 0.5 | 9.1 | 4 |
| 120 | 0.8 |  | 0.8 |  | 0.8 |  | 0.2 |  | 0.8 |  | 0.8 |  |
| 120 | 100.8 |  | 100.8 |  | 100.8 |  | 20.2 |  | 100.8 |  | 100.8 |  |
| 123 | 55.3 | 32 | 55.3 | 2 | 55.3 | 32 | 11.1 | 8 | 55.3 | 16 | 55.3 | 32 |
| 126 | 30.4 | 16 | 30.4 | 0 | 30.4 | 8 | 6.1 | 4 | 30.4 | 4 | 30.4 | 16 |
| 132 | 9.1 | 2 | 9.1 | 0 | 9.1 | 1 | 1.8 | 0 | 9.1 | 0.5 | 9.1 | 4 |
| 144 | 0.8 | 0 | 0.8 | 0 | 0.8 | 0 | 0.2 | 0 | 0.8 | 0 | 0.8 | 0 |

* All concentrations are reported as multiples of the MICs reported in the Results. THY = Predicted, BIO = Bioassay determined.
