# Supplementary material for: Staphylococcus aureus in Continuous Culture: A Tool for the Rational Design of Antibiotic Treatment Protocols
Source: PLoS One. 2012 Jul 20;7(7):e38866. doi: 10.1371/journal.pone.0038866 (PMC3401188; doi:10.1371/journal.pone.0038866)
Supplement: Appendix S1 — Mathematical equations describing the pharmacokinetics, PK, of antibiotics in continuous cultures. (DOCX) [file pone.0038866.s003.docx]

**APPENDIX S1**

The pharmacokinetics, PK, of antibiotics in continuous cultures

As long as the sum of the rate of washout and decay in the effective concentration of an antibiotic (δ = w + d) hr ^-1^, remains constant, the rate of change in the concentration of this drug, A mg/L, between doses will be given by,

$\frac{dA}{dt}= -\delta A$

In this model, the peak MIC is the maximum level, which is that of the dose A(0), assuming no residual drug. The time above the MIC can be calculated as,

$$T=\frac{1}{\delta}\ln\left( \frac{A_{0}}{\mathrm{MIC}} \right)$$

and the area under the antibiotic concentration / time curve is

$$AUC= \frac{A_{0}}{2} ( e^{\delta t_{1}}- e^{\delta t_{2}})$$

where t_1_ is the time the dose is given and t_2_ the time the next dose is administered, where t_2_ is sufficiently great for the concentration to be effectively 0.

24 hours in the experiments depicted in Figure 2 are more than sufficient.
